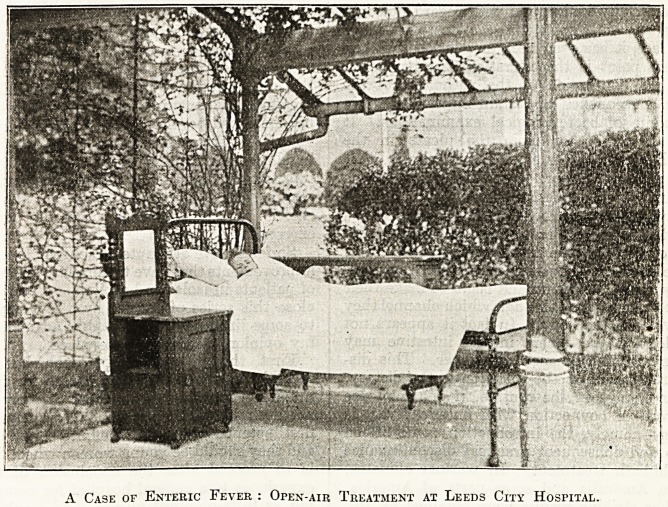# The Treatment of Enteric Fever

**Published:** 1912-09-14

**Authors:** A. Knyvett Gordon

**Affiliations:** formerly Medical Superintendent of Monsall Hospital and Lecturer on Infectious Diseases in the University of Manchester.


### SOME FEVER HOSPITALS AND THEIR WORK.*

### VII.—The Treatment of Enteric Fever.

By A. KNYVETT GORDON, M.B. Cantab., formerly Medical Superintendent of Monsall Hospital and Lecturer on Infectious Diseases in the University of Manchester.

We may now leave the subject of diphtheria and pass to another of the diseases which are usually admitted to fever hospitals—namely, enteric or typhoid fever.

Though there has been nothing in connection with the treatment of this disease so striking as the introduction of antitoxic serum, there has yet been a steady diminution in its mortality in those hospitals where the treatment of the patients—as apart from their isolation—is taken seriously, and it may be as well to inquire on what this depends.

Omitting any discussion of the question of the severity of the cases in different epidemics, there can be no doubt that much of this improvement is due to a change which has taken place in our conception of the pathology of the disease and the effect which this has had on treatment.

A Case of Enteric Fever: Open-air Treatment at Leeds City Hospital.

Formerly enteric fever was regarded as an ulceration of the intestine, and as little else. The characteristic bacilli were known to be present in the ulcers, and it was assumed that the affected length of intestine was *the* site of the disease, and that the poisonous products of the bacilli to which the fever and other constitutional symptoms are due were produced there, and, practically speaking, there alone. Consequently it was taught that the diarrhœa with which the disease was then so often associated was due to ulceration of the bowel, and also that (in order not to irritate the ulcerated surface by the passage over it of undigested solid food) nothing but milk should be given by the mouth until the temperature had fallen to the normal, and often for a fortnight subsequently. It was held that any attempt to give anything but milk would involve the risk of hæmorrhage from the ulcers or of perforation of the bowel itself.

Inasmuch as the temperature in enteric fever commonly keeps up for a period of three weeks or so, considerable emaciation took place, and the patient subsequent to the fall of the temperature inevitably went through a protracted convalescence held to be characteristic and inevitable.

In this conception of enteric fever there are, however, two fallacies. To take them in the order in which they became apparent we have first the fact that the "typical" diarrhœa with its "characteristic" stools was due not entirely, or even mainly, to enteric fever, but to the inability of the patient to digest milk for the prolonged period during which it was the custom to administer it. The "typical" typhoid stools always contain curds of undigested milk, and it has been found that when the milk is stopped the "typhoid" stools disappear also. We also have the fact that these stools appear in other diseases, notably in some cases of septic scarlet fever, when we inadvertently give the patient more milk than he can digest.

Now, when the diarrhœa ceases, as it practically

* Previous articles appeared on July 13, 20, Aug. 10, 17, 24, 31.

always does in the normal case when a suitable diet has been substituted for the milk—I should add that nearly all the cases that are admitted into fever hospitals are found to have been fed on milk alone previous to their reception—it is found that the majority of the patients become hungry, even though the temperature keeps up. Acting on this indication it has been also found that, if they can digest some kind of soft solid food, not only is the mortality during the pyrexial period considerably lessened, but the time of convalescence is shortened and the incidence of the various complications is also diminished, notably of those—namely, hæmorrhage from and perforation of the bowel, and relapse of the disease, all of which the limitation of the patient to an exclusively milk diet was supposed to render more frequent.

Speaking generally, it is the custom nowadays in most fever hospitals to vary the diet in enteric fever, even during the pyrexial period, and to include soft solid food in the daily menu. Of course, the golden rule in enteric fever—namely, to treat each patient individually, and with regard not only to the disease, but also to his reaction to it—holds good; in fact, it has even been emphasised by this.

The next point is the alteration that has taken place in our conception of the pathology of the disease in consequence of certain improvements in the technique of bacteriological examinations. It has been found so far from the ulcers in the intestine being the main site of the bacteria, that these are present in much greater numbers in the upper part of the small intestine than in the ulcerated portion lower down. Also, the organisms are to be found in the circulating blood in the great majority of cases in the first week of the disease, and as the illness progresses they are discovered less frequently in that situation and begin to make their appearance in the urine, through which channel they ultimately leave the body. In fact it appears not improbable that the ulcers in the intestine may be a secondary phenomenon altogether. This discovery further justifies the variation in the dietary.

Another change in the routine, if one may use such a word in connection with enteric fever, is interesting—namely, the introduction some fifteen years ago and subsequent practical discontinuance of the cold-bath treatment. In warmer climates, notably in Australia and some parts of America, some rather striking results were obtained by frequent immersion of the patient during the pyrexial period in a cool or cold bath, and the method was subsequently tried extensively in this country. It was found, however, that while the cool bath was undoubtedly beneficial to a certain type of robust patient, yet the results in most cases did not come up to the expectations that had been formed of its value from the foreign statistics. As a routine practice the cold bath is virtually now extinct, as it has been found that one of its advantages—namely, an increased elimination through the skin of toxic substances—can be obtained more easily and with less risk to the patient by administering large quantities of water by the mouth, which causes the toxins to leave the body through the kidneys instead.

Another improvement is the treatment of perforation of the intestine on surgical lines by opening of the abdomen and suture of the perforated ulcer directly a diagnosis of this occurrence has been made. This is another reason for the provision of an operating theatre in the modern fever hospital, as it is manifestly impossible to move the patient with safety to a general hospital for operation. The results of operations are fairly good when one considers the fact that all the patients are in a weak state from the attack of enteric fever itself, apart from the shock due to the perforation of the bowel. About one-fifth of the cases recover when thus treated, while it is universally admitted that in any given case where perforation has undoubtedly taken place the chance of recovery without operation is practically non-existent.

Inasmuch as the urine and stools of a typhoid patient are highly infectious to others, it is not desirable that they should be consigned to the ordinary drainage system of a city without previous sterilisation. When this can be done mechanically by boiling of the excreta in a special plant, as at the Leeds City Hospital, it is a distinct advantage to the public health that the town's sewage should be preserved from contamination. When, however, the only alternative is the chemical disinfection of the stools by the nurses it is better that they should be consigned to the drains immediately they are voided by the patient as the necessary prolonged handling of the utensils, etc., generally results in infection of the nursing staff. In my view, rubber gloves should always be worn by the nurses in enteric wards whenever there is contact with excreta.

So far I have attempted to chronicle a few of the improvements that have taken place in the treatment of patients in isolation hospitals. I now propose to close this series of articles by a few suggestions as to some lines on which further advance might, in my opinion, profitably take place.

First, though much, very much, has been done recently in the training of nurses for fever work, much yet remains to be done. In my view it is not desirable that nurses should be engaged in the first instance except for a definite period of training, and they should be young women whose intention it is to proceed after the conclusion of their fever experience to a general hospital for complete training as fully-qualified nurses. As a rule it is quite safe to accept probationers at a fever hospital at the age of twenty-one, which is two years younger than the limit at most general hospitals. Provided that the instruction given at the fever hospital is on well-recognised scientific lines—and it is grossly unfair to the nurses if it is not—the general hospitals are usually glad to have them as probationers subsequently, and my experience has been that they do very well there, and that their previous fever experience is a distinct advantage. The fact is that, when one comes to think of it, all the precautions which are taken in a fever hospital to diminish mortality and to prevent cross-infection resolve themselves ultimately—though structural facilities may help—into asepsis on the part of the nurses. And asepsis is like golf: it must be learnt young! It is also most important that the sisters of the wards shall have received a full period of general training and should themselves be capable of instructing probationers.

Another point is that practically all fever hospitals are understaffed, even when only the average number of beds is occupied. When the "extra beds" in each ward are occupied also the pressure on the nursing becomes dangerous in that it renders it impossible to "isolate," "barrier," or otherwise give special treatment to many cases which ought really to receive it. And very many hospital committees object more strongly to expenditure on nursing than to outlay in almost any other department—and yet nursing is nowadays *the* vital point.

Then our point of view as to the kind of disease that requires isolating seems to be shifting somewhat. While I do not for one moment hold with those who would have us believe that we may as well cease to admit cases of scarlet fever to our hospitals at all for all the good it has been to the community, yet the view seems to be gaining ground that cases of phthisis, at all events, and perhaps of measles and whooping-cough also, should be admitted, and mild cases of scarlet fever, when the accommodation in the house is fairly good, be left at home. If this be done the difficulty of separating the cases in the hospital increases, and the problem has to be solved either by building more isolation wards or by the barrier or bed isolation system. In any case we come again to the necessity for more nurses and for increased perfection in aseptic technique.

It will probably always be considered advisable to admit severe cases of scarlet fever and also even mild cases from houses where anything resembling isolation is obviously impossible, but the omission of the remainder would leave a good deal of room for the extension of the municipal treatment of phthisis, which appears to be an urgent need.

There is yet another point in which, in my opinion, fever hospitals might extend their usefulness. Many cases of deafness in childhood, and not a few of deaf-mutism, are due to scarlet fever, and it is certainly a question whether the ears of those suffering from scarlet fever receive adequate attention in our fever hospitals. There is too much of the tendency to regard discharging ears only from the point of view of their infectivity, and to treat them *en bloc* by irrigation with various stock lotions rather than by the measures (some of which are surgical in character) which would be found to be suitable to the individual patient after careful examination such as he would receive in the outpatient clinique of a skilled otologist. When the necessary skill is available this should obviously be undertaken by the resident medical staff of the fever hospital itself, and I am strongly of opinion that previous experience in otology should be demanded of all candidates for the post of resident medical officer to a fever hospital. When, however, for any reason, this is not possible, a consulting otologist should be appointed, who should examine all the patients with discharging ears at frequent intervals, and be provided by the authorities with every facility for carrying out any surgical measures that may be required. By this means the patients would receive adequate attention at the time when treatment is of most avail, instead of being left to attend the outpatient clinique some time after the mischief has been done. There is, moreover, the undoubted fact that every person with a chronic discharging ear is liable at any time to the risk of death or very serious illness from extension of the trouble beyond the ear itself to the brain or its membranes. No patient should be, or for that matter need be, discharged from a fever hospital with a running ear.

These are, however, but suggestions for the future and do not invalidate the contention that I have endeavoured to make—namely, that the modern fever hospital is an institution to which no parent need be afraid of trusting the care of a child suffering from a notifiable infectious disease, and that so far from being content merely with isolating the patient for the benefit of the community, every care is taken in the vital matter of safeguarding him from the risk of contracting another infectious disease.

## Figures and Tables

**Figure f1:**